# Therapeutic Challenges in Patients with Gynecologic Carcinosarcomas: Analysis of a Multicenter National Cohort Study from the French Prospective TMRG Network

**DOI:** 10.3390/cancers14020354

**Published:** 2022-01-12

**Authors:** Clémence Romeo, Olivia Le Saux, Margaux Jacobs, Florence Joly, Gwenael Ferron, Laure Favier, Jean-David Fumet, Nicolas Isambert, Pierre-Emmanuel Colombo, Renaud Sabatier, Ludovic Bastide, Amandine Charreton, Mojgan Devouassoux-Shisheboran, Witold Gertych, Coraline Dubot, Diana Bello Roufai, Guillaume Bataillon, Dominique Berton, Elsa Kalbacher, Patricia Pautier, Christophe Pomel, Caroline Cornou, Isabelle Treilleux, Audrey Lardy-Cleaud, Isabelle Ray-Coquard

**Affiliations:** 1 Medical Oncology Department, Centre Léon Bérard, 69008 Lyon, France; Amandine.charreton@lyon.unicancer.fr (A.C.); Isabelle.ray-coquard@lyon.unicancer.fr (I.R.-C.); 2 Cancer Research Center of Lyon CRCL, UMR Inserm 1052, CNRS 5286, Centre Léon Bérard, 69008 Lyon, France; Olivia.lesaux@lyon.unicancer.fr; 3 Medical Oncology Department, Centre François Baclesse, 14000 Caen, France; M.jacobs@baclesse.unicancer.fr (M.J.); F.joly@baclesse.unicancer.fr (F.J.); 4 GINECO Group, 75008 Paris, France; Ferron.Gwenael@iuct-oncopole.fr (G.F.); LFavier@cgfl.fr (L.F.); NIsambert@cgfl.fr (N.I.); Pierre-Emmanuel.Colombo@icm.unicancer.fr (P.-E.C.); Sabatierr@ipc.unicancer.fr (R.S.); Mojgan.devouassoux@chu-lyon.fr (M.D.-S.); Coraline.dubot@curie.fr (C.D.); Guillaume.bataillon@curie.fr (G.B.); Dominique.berton@ico.unicancer.fr (D.B.); Ekalbacher@chu-besancon.fr (E.K.); Patricia.pautier@gustaveroussy.fr (P.P.); Christophe.pomel@clermont.unicancer.fr (C.P.); Isabelle.treilleux@lyon.unicancer.fr (I.T.); 5 Department of Surgical Oncology, Institut Claudius Regaud-IUCT, 31100 Toulouse, France; 6 Medical Oncology Department, Centre Georges François Leclerc, 21000 Dijon, France; Jdfumet@cgfl.fr; 7 Surgical Oncology Department, Institut du Cancer de Montpellier, 34090 Montpellier, France; 8 Medical Oncology Department, Institut Paoli Calmettes, 13009 Marseille, France; BASTIDEL@ipc.unicancer.fr; 9 Tumor Biology Department, Centre Hospitalier Lyon-Sud, Hospices Civils de Lyon, 69495 Lyon, France; 10 Gynaecologic Surgery Department, Hôpital Lyon Sud, Hospices Civils de Lyon, 69495 Lyon, France; witold.gertych@chu-lyon.fr; 11 Medical Oncology Department, Institut Curie, Saint-Cloud, 92210 Paris, France; diana.belloroufai@curie.fr; 12 Tumor Biology Department, Institut Curie, 75005 Paris, France; 13 Medical Oncology Department, Institut de Cancérologie de l’Ouest, 44800 Saint Herblain, France; 14 Medical Oncology Department, CHRU de Besançon, 25000 Besançon, France; 15 Medical Oncology Department, Gustave Roussy, 94805 Villejuif, France; 16 Surgical Oncology Department, Centre Jean Perrin, 63011 Clermont-Ferrand, France; Caroline.cornou@clermont.unicancer.fr; 17 Tumor Biology Department, Centre Léon Bérard, 69008 Lyon, France; 18 Biostatistic Department, DRCI, Centre Léon Bérard, 69008 Lyon, France; Audrey.lardy-cleaud@lyon.unicancer.fr; 19 University Claude Bernard Lyon 1, 69100 Villeurbanne, France

**Keywords:** ovarian carcinosarcoma, uterine carcinosarcoma, rare cancer, adjuvant treatment, chemotherapy, cytotoxic agent, TMRG network

## Abstract

**Simple Summary:**

Gynecologic carcinosarcomas are rare and aggressive diseases with a poor prognosis. The rarity of these tumors explains the lack of robust and specific data available in the literature. Using the data from the French National Rare Malignant Gynecological Tumors (TMRG) network, we conducted a multicentric cohort study to explore several burned questions. The main objective was to assess the outcome of patients with carcinosarcomas recorded in the network and to investigate the efficacy of initial adjuvant treatment and recurrent therapeutic strategies in a real-life setting. Four hundred and twenty-five patients were analyzed including 313 uterine and 112 ovarian carcinosarcomas. Our data suggest positive impact of adjuvant chemotherapy on survival in all stages (including FIGO IA uterine carcinosarcomas) and the importance of platinum-based combination for the treatment of relapse. In addition we report median PFS for various therapeutic strategies in the relapse setting.

**Abstract:**

Background: Gynecological carcinosarcomas are rare and aggressive diseases, with a poor prognosis. The rarity of these tumors explains the lack of robust and specific data available in the literature. The objective of this study was to investigate the impact of initial adjuvant treatment and recurrent therapeutic strategies. Patients and methods: A multicentric cohort study within the French national prospective Rare Malignant Gynecological Tumors (TMRG) network was conducted. Data from all included carcinosarcomas diagnosed between 2011 and 2018 were retrospectively collected. Results: 425 cases of uterine and ovarian carcinosarcomas (*n* = 313 and *n* = 112, respectively) were collected and analyzed from 12 participating centers. At diagnosis, 140 patients (48%) had a FIGO stage III–IV uterine carcinosarcoma (UCS) and 88 patients (83%) had an advanced ovarian carcinosarcoma (OCS) (FIGO stage ≥ III). Two hundred sixty-seven patients (63%) received adjuvant chemotherapy, most preferably carboplatin-paclitaxel regimen (*n* = 227, 86%). After a median follow-up of 47.4 months, the median progression-free survival (mPFS) was 15.1 months (95% CI 12.3–20.6) and 14.8 months (95% CI 13.1–17.1) for OCS and UCS, respectively. The median overall survival for OCS and UCS was 37.1 months (95% CI 22.2–49.2) and 30.6 months (95% CI 24.1–40.9), respectively. With adjuvant chemotherapy followed by radiotherapy, mPFS was 41.0 months (95% CI 17.0–NR) and 18.9 months (95% CI 14.0–45.6) for UCS stages I–II and stages III–IV, respectively. In the early stage UCS subgroup (i.e., stage IA, *n* = 86, 30%), mPFS for patients treated with adjuvant chemotherapy (*n* = 24) was not reached (95% CI 22.2–NR), while mPFS for untreated patients (*n* = 62) was 19.9 months (95% IC 13.9–72.9) (HR 0.44 (0.20–0.95) *p* = 0.03). At the first relapse, median PFS for all patients was 4.2 months (95% CI 3.5–5.3). In the first relapse, mPFS was 6.7 months (95% CI 5.1–8.5) and 2.2 months (95% CI 1.9–2.9) with a combination of chemotherapy or monotherapy, respectively (*p* < 0.001). Conclusions: Interestingly, this vast prospective cohort of gynecological carcinosarcoma patients from the French national Rare Malignant Gynecological Tumors network (i) highlights the positive impact of adjuvant CT on survival in all localized stages (including FIGO IA uterine carcinosarcomas), (ii) confirms the importance of platinum-based combination as an option for relapse setting, and (iii) reports median PFS for various therapeutic strategies in the relapse setting.

## 1. Introduction

Gynecologic carcinosarcomas (CS) are rare and aggressive tumors that have an incidence of approximately 5% of all uterine cancers and 1–3% of all malignant ovarian tumors [1,2,3]. Carcinosarcomas are biphasic neoplasms composed of both high-grade malignant epithelial and mesenchymal elements. It is now acknowledged that carcinosarcoma’s origin is monoclonal and that it arises as a result of dedifferentiation of the carcinoma component [4,5,6,7]. This could partially explain the natural history of CS, which is more similar to carcinomas than to sarcomas in terms of dissemination and sensitivity to cytotoxic agents [4,8]. Hence, uterine carcinosarcoma (UCS) has been classified as an endometrial carcinoma in the 2003 World Health Organization (WHO) classification of tumors of the female genital tract, while it was previously considered as a malignant mixed mullerian tumor [9].

The clinical behavior of CS is very aggressive, and its prognosis is poor compared with high-grade endometrial and ovarian carcinoma [10,11,12]. Advanced disease at the time of diagnosis and the frequent local and distant recurrences may explain the low five-year survival rate <30% [12,13,14,15].

Optimal cytoreductive surgery is the cornerstone of treatment [16], but the overall recurrence rate of 60% [14,17] underlines the need for effective adjuvant- and relapse-therapeutic strategies. Given the low incidence of these tumors, prospective trials of chemotherapeutic approaches have been difficult to perform. Data guiding chemotherapy is largely extrapolated from retrospective studies and experience in epithelial cancers [16]. Although adjuvant pelvic radiotherapy may decrease UCS pelvic recurrence [18,19,20,21,22], no adjuvant therapy (chemotherapy, external radiotherapy, or brachytherapy) has specifically demonstrated a significant improvement in overall survival (OS) [16]. Hence, optimal adjuvant therapy is still debated. Carboplatin-paclitaxel is the preferred regimen compared to ifosfamide-based combination due to a manageable safety profile and comparable progression-free survival (PFS). This was reported in the phase III NRG Oncology clinical trial GOG-0261, which compared carboplatin-paclitaxel with paclitaxel-ifosfamide in chemotherapy-naive patients with stage I–IV, persistent, or recurrent carcinosarcoma of the uterus or ovary [23]. Few data are available concerning subsequent lines of chemotherapy in advanced disease.

Considering the limited data available in the literature, we analyzed our national prospective database to explore several crucial questions for CS: Is adjuvant therapy beneficial in early stage UCS? What is the best option in the relapse setting in terms of systemic therapies?

The objective of the current study was to assess the outcome of patients with carcinosarcomas recorded in the Rare Malignant Gynecological Tumors (TMRG) network and to investigate the efficacy of initial adjuvant treatment and recurrent therapeutic strategies in a real-life setting.

## 2. Patients and Methods

### 2.1. Patients and Data Collection

The French TMRG powered by the ARCAGY-GINECO group has provided, since 2011, a national prospective network supporting diagnosis and management for all rare gynecological cancers. The goals of this network are to include systematic double pathology review by an expert in gynecological malignancies and to provide multidisciplinary expert advice on the management of these tumors [24]. National Clinical Practice Guidelines are available and regularly updated on the network website (www.ovaire-rare.org, accessed on 1 November 2021). The network also aims to build a unique database, gathering all cases of rare gynecological tumors diagnosed in France that could be used for the purpose of scientific studies [25,26].

We carried out a retrospective national multicenter cohort analysis within the TMRG network. Women above 18 years of age with a diagnosis of uterine or ovarian carcinosarcoma, histologically confirmed with double pathology review by an expert in gynecological malignancies, treated between January 2011 and December 2018, were identified from the TMRG database. Each patient provided written informed consent; data were anonymized and registered in the national database. Data were retrospectively extracted from medical records and included demographic and clinicopathologic features, treatment, and outcome and follow-up information. Patients’ initial characteristics consisted of age at diagnosis, year of diagnosis, personal and familial history of cancer, use of anterior tamoxifen, use of menopausal hormone therapy and prior pelvic radiation exposure, FIGO stage (using the International Federation of Gynecology and Obstetrics FIGO classification 2009 or 2014), and metastatic site location. *BRCA* mutational status were also collected. Histological type was recorded and defined according to the WHO classification [9]. The carcinoma components were grouped into endometrioid, serous, and other (i.e., clear cell, undifferentiated, and mixed histology subtypes). The sarcoma components were divided into homologous (i.e., undifferentiated round cell or spindle cell sarcomatous proliferation with some features similar to endometrial stromal sarcoma, leiomyosarcoma, or fibrosarcoma) and heterologous (i.e., cartilaginous, rhabdomyosarcomatous, osteosarcomatous, or liposarcomatous differentiation elements). Information regarding treatment, including surgery, chemotherapy and radiation therapy (external radiotherapy and/or brachytherapy), and dates of progression and death were collected.

Quality of surgery was defined by the completeness of cytoreduction score (CC-score) and the score using resection margins (R0 to R2) for OCS and UCS, respectively [27]. Surgery was considered as macroscopically complete in cases of CC-0 or R0. Chemotherapy regimens in the first relapse setting were classified into eight subgroups: platinum/paclitaxel-based chemotherapy; platinum/anthracycline-based chemotherapy; platinum-free anthracycline-containing combination; platinum/gemcitabine-based chemotherapy; anthracycline monotherapy; platinum monotherapy; platinum-free and anthracycline-free monotherapy; and innovative therapy. In second relapse, chemotherapy regimens were classified into eight subgroups: platinum/paclitaxel-based chemotherapy; anthracycline-containing combination; anthracycline monotherapy; anthracycline-free combination; gemcitabine monotherapy; taxane monotherapy; innovative therapy; and other regimens.

In order to analyze specifically the outcome of patients with gynecological carcinosarcoma, an authorization was obtained from the French data protection authority (CNIL) on January 2019.

### 2.2. Statistical Plan

Descriptive statistics were used to summarize patients’ initial characteristics. Overall survival was calculated from the date of diagnosis to the date of death or censored to the date of the latest news. Progression-free survival (PFS) was calculated from the date of diagnosis to the date of the first event, defined as relapse, progressive tumor, or death from any cause or censored to the date of the latest news. PFS of subsequent systemic therapy for recurrent/metastatic cancer was calculated from start of the first systemic chemotherapy regimen for recurrent/metastatic cancer to the date of event, defined as relapse, progressive tumor, or death from any cause or censored to the date of the latest news. Survival curves with associated log-rank tests were generated using the Kaplan–Meier method. Median follow-up was calculated using reverse Kaplan–Meier estimation. Univariate and multivariate Cox proportional hazards models were performed to identify potential prognostic factors such as age, FIGO stage, and systemic therapies. Only sufficiently informative variables (less than 10% of missing data) with *p* < 0.10 on the univariate analysis were included in the multivariable model. A stepwise backward selection with a *p* = 0.05 threshold was used to obtain the final multivariable model. Hazard Ratios (HRs) are presented with 95% confidence intervals (CI).

All statistical analyses were performed using SAS (version 9.4 SAS Institute Inc., Cary, NC, USA).

## 3. Results

### 3.1. Patient Characteristics

A total of 425 patients diagnosed with gynecological carcinosarcomas (uterine, *n* = 313, and ovarian, *n* = 112), were identified from the TMRG database in 12 centers from January 2011 to December 2018. Patient characteristics are summarized in Table 1. The median age at diagnosis was 69 years (range, 26 to 90 years). Most of the patients with ovarian carcinosarcoma (OCS) had an advanced disease with FIGO stage ≥ III (*n* = 88, 83.0%) at diagnosis. One hundred sixty-eight (54.5%) and 140 (45.4%) patients with UCS had a FIGO stage I/II versus III/IV, respectively. Forty-two patients (13.6%) had stage IV UCS at diagnosis.

Concerning histological subtypes, 147 patients (55.1%) had a major epithelial component at diagnosis (*n* = 158, 37.2% missing data). The epithelial component was predominantly composed of serous adenocarcinoma (*n* = 50, 65.8%) in OCS, and endometrioid carcinoma (*n* = 99, 47.6%) in UCS. The sarcoma component consisted of heterologous (*n* = 224, 64.7%) and homologous (*n* = 122, 35.3%).

*BRCA* gene screening was performed for 40 patients with primary OCS (35.7%) and 13 patients with UCS (4.2%). Both germline and somatic testing were performed for 30 patients (56.6%), while 14 patients (26.4%) had only germline testing and 9 patients (16.9%) only somatic testing. Deleterious mutations in *BRCA* genes were found in 5 out of 53 patients (9.4%; *n* = 4/40 OCS and *n* = 1/13 UCS) and were all found in germline testing; 2 of them were in *BRCA1* (including the patient with UCS) and 3 were in *BRCA2*.

### 3.2. Initial Treatment Characteristics and Outcome

The majority of patients underwent surgery (*n* = 383/425, 90.3%), with R0 resection for 88.6% of UCS (*n* = 195) and CC-0 score for 77.4% of OCS patients (*n* = 65). For OCS patients, both upfront primary debulking and interval cytoreductive surgery were performed (*n* = 61/97, 62.9% and *n* = 36/97, 37.1%, respectively), while the great majority of UCS had an upfront surgery (*n* = 272/286, 95.1%).

Two hundred sixty-seven patients (63.3%) received initial chemotherapy (CT) including 45 (16.8%) for FIGO stage IV. Almost all OCS (*n* = 103/112, 92.8%) and only 52.7% of UCS (*n* = 164/313) received CT. Median number of cycles was 6 (range 1–12). Carboplatin-paclitaxel (CP) regimen was administered to 227 patients (85.0%). Only 7 patients (2.6%) received a combination based on ifosfamide. Concerning UCS, adjuvant chemotherapy was administered to 24/86 (27.9%) stage IA, 57/168 (33.9%) stage I–II, and 106/138 (76.8%) stage III–IV patients. One-third of patients with OCS (*n* = 32, 31.1%) received a combination of chemotherapy with antiangiogenic therapy.

Adjuvant pelvic radiotherapy (RT) was performed for 207 patients (66.1%) with an UCS, of which 121/140 (86.4%) were stage I (including 72/86 stage IA), 20/28 (71.4%) stage II, 58/98 (59.2%) stage III, and 5/42 (11.9%) stage IV patients. External RT was administered with subsequent brachytherapy for 157 UCS patients (76.2%).

**Figure 1:**
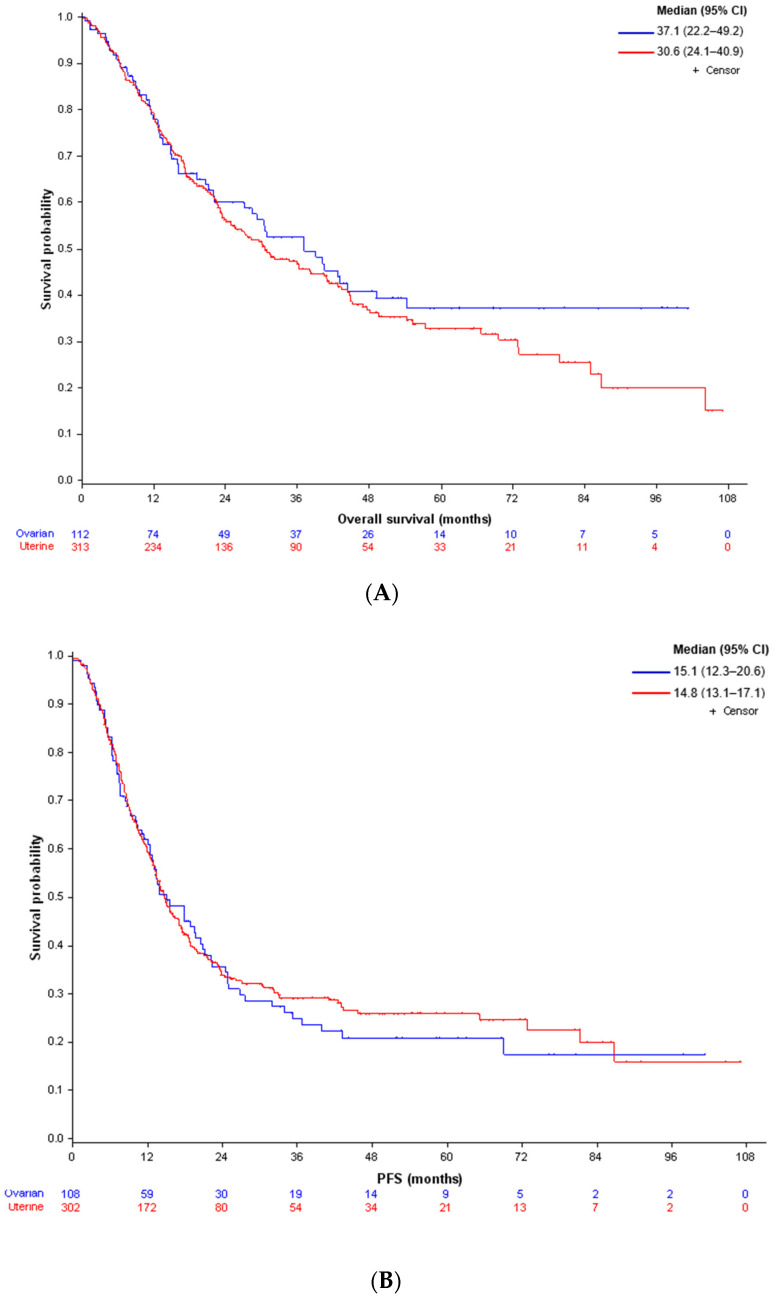
(**A**) Kaplan–Meier curves for overall survival (OS) in UCS (red curve) and OCS (blue curve). (**B**). Kaplan–Meier curves for progression-free survival (PFS) in UCS (red curve) and OCS (blue curve).

Median follow-up duration was 47.4 months (range, 1.4–106.9). The median overall survival (OS) was 37.1 months (95% CI 22.2–49.2) and 30.6 months (95% CI 24.1–40.9) for OCS and UCS, respectively (Figure 1A). The median progression-free survival (PFS) was 15.1 months (95% CI 12.3–20.6) and 14.8 months (95% CI 13.1–17.1) for OCS and UCS, respectively (Figure 1B).

Median PFS according to stage is described in Table 2 and Table 3 for OCS and UCS, respectively.

### 3.3. Front-Line Management

In the UCS subgroup, regardless of stage, there was no significant difference in PFS between chemotherapy-treated (*n* = 161) and untreated patients (*n* = 140) (mPFS 15.6 months (95% CI 13.1–18.3) and 14.0 months (95% CI 10.9–17.8), respectively; HR 0.91 (0.69–1.19) *p* = 0.4809, logrank test). Comparison of different adjuvant strategies (adjuvant CT alone, RT alone, concomitant chemoradiotherapy, CT followed by RT, or no adjuvant therapy) for early and advanced-stage are described in Table 4 and Table 5. A longer PFS was observed with sequential combined adjuvant therapy (mPFS 41.0 months (95% CI 17.0–NR) for stage I–II; and mPFS 18.9 months (95% CI 14.0–45.6) for stage III–IV).

**Figure 2:**
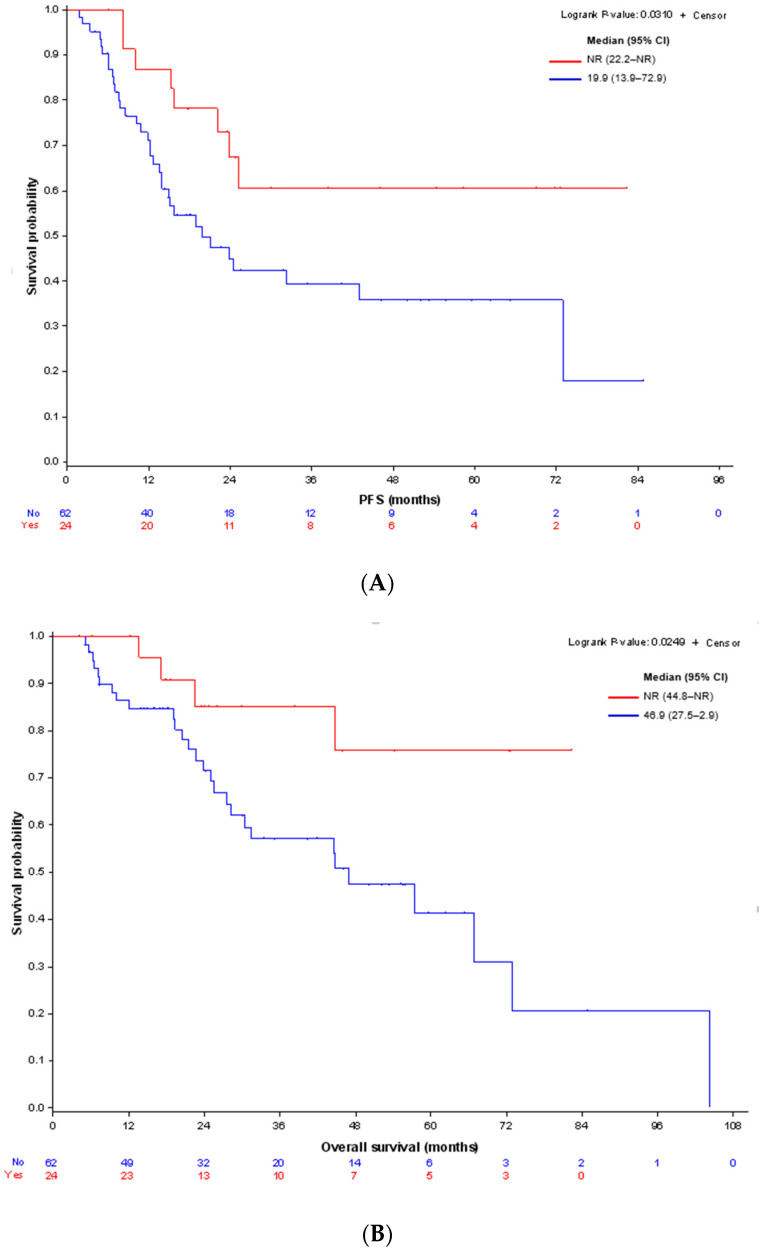
(**A**) Kaplan–Meier curves for PFS in stage IA UCS according to adjuvant chemotherapy. (**B**) Kaplan–Meier curves for OS in stage IA UCS according to adjuvant chemotherapy.

In patients with stage IA UCS (*n* = 86), median PFS for patients treated with adjuvant chemotherapy (*n* = 24) was not reached (NR) (95% CI 22.2–NR), while mPFS for patients who did not receive adjuvant chemotherapy (*n* = 62) was 19.9 months ((95% IC 13.9–72.9), HR 0.44 (0.20–0.95) *p* = 0.0310, logrank test) (Figure 2A). This benefit was also found in OS (mOS NR (95% IC 44.8-NR) and 46.9 months (95% IC 27.5–72.9), respectively) with a hazard ratio of 0.32 (0.11–0.91) for patients with chemotherapy, *p* = 0.0249, logrank test (Figure 2B). In this subgroup, 40 patients relapsed (46.5%), including 29 patients (72.5%) previously treated with RT and 32 patients (80%) who did not receive adjuvant chemotherapy.

Multivariate analysis of PFS in the subgroup of UCS revealed that FIGO stage and initial radiotherapy were significantly associated with PFS (Table 6). PFS was better in stage I patients versus stage II, III, or IV patients and in patients with radiotherapy.

In the OCS subgroup, almost all patients had first line chemotherapy (*n* = 103, 92.8%) either neoadjuvant (*n* = 45, 43.7%), adjuvant (*n* = 52, 50.5%), or metastatic (*n* = 6, 5.8%). No significant difference was observed in PFS between patients treated or untreated with concomitant bevacizumab (mPFS 17.9 months (95% CI 11.3–22.1) and 15.1 months (95% CI 11.0–24.8), respectively; adjusted on stage HR 0.983 (0.576–1.6.76) *p* = 0.9496).

Multivariate analysis of PFS in the subgroup of OCS revealed that surgery (*p* < 0.0001) and front-line chemotherapy (*p* = 0.0103) were significantly associated with a better PFS (Table 7).

### 3.4. Recurrence or Relapse Setting

At the time of analysis, 264 of the 412 evaluable patients (64.1%, *n* = 73/108 OCS and *n* = 191/304 UCS) had a recurrence or progression of the disease, among which 174 (65.9%) were initially treated with chemotherapy at diagnosis. Median time of relapse after the end of chemotherapy was 5.2 months (range −0.7–61.4). In the relapse setting, median number of therapeutic lines was 1 (range 0−7), and 194 patients (73.5%) received at least one line. PFS was not evaluable in nine patients. One hundred thirteen (61.1%) and 72 (38.9%) out of 185 evaluable patients were treated with a combination of chemotherapy and monotherapy, respectively. The median PFS (mPFS) at the first relapse was 4.2 months (95% CI 3.5–5.3); 4.8 months (95% CI 2.8–8.5) in OCS and 4.1 months (95% CI 3.1–5.2) in UCS, respectively. In the overall population, mPFS with a combination of chemotherapy was 6.7 months (95% CI 5.1–8.5) versus 2.2 months (95% CI 1.9–2.9) with monotherapy, *p* < 0.001. This benefit was maintained in the subgroup of patients pretreated by chemotherapy at diagnosis (mPFS 7.8 months (95% CI 4.8–10.4) and 2.2 months (95% CI 1.8–2.9), respectively, *p* < 0.001). There was no significant difference between combination regimens and monotherapy in subgroups according to the time of relapse after initial chemotherapy (<6 or ≥6 months). In the subgroup of patients relapsing ≥ 6 months after initial chemotherapy, mPFS was 10.4 months (95% CI 7.2–12.4) and 3.1 months (95% CI 1.3–14.0) for combination regimen and monotherapy, respectively, *p* = 0.8382.

Overall response rates (ORRs) of the different agents ranged from 0% to 62.5% and are described in Table 8. Median PFS durations according to chemotherapy regimens are reported in Table 9.

Among patients presenting with first relapse, almost all patients had a second relapse or progression (*n* = 152/185, 82.2%; with 42 OCS and 110 UCS) and 95 (62.5%) had a new therapeutic line (*n* = 28 OCS and *n* = 67 UCS). The distribution of chemotherapy regimens was as follows: 20% of anthracycline monotherapy (*n* = 19), 11.6% of platinum/paclitaxel-based chemotherapy (*n* = 11), 12.6% of anthracycline-containing combination (*n* = 12), 10.5% of innovative therapy (*n* = 10), 9.5% of gemcitabine monotherapy (*n* = 9), 10.5% of taxane monotherapy (*n* = 10), 6.3% of anthracycline-free combination (*n* = 6), 17.9% of others regimens and one unknown.

Median PFS at second systemic line/relapse was 4.4 months (95%IC 1.9–6.4) in OCS and 2.4 months (95%IC 1.8–3.0) in UCS. Median PFS and ORRs according to chemotherapy regimens are reported in Table 10 and Table 11, respectively.

Among all patients, five patients (4.5%) with OCS and one patient (0.3%) with UCS received immunotherapy in a clinical trial at first or second relapse. One patient with OCS (0.9%) received PARP inhibitor maintenance therapy after response to platinum-based chemotherapy.

## 4. Discussion

In this multicenter national cohort study, we collected retrospective data from 425 patients with gynecological carcinosarcomas within the French prospective TMRG network. This study has shown that, in the adjuvant setting, multimodal treatment strategy appears more efficient for uterine carcinosarcomas, and chemotherapy significantly improves survival in early stage IA. In the relapse setting, while survival outcomes are poor, combination chemotherapy increases ORR and improves progression-free survival, even in pre-treated patients.

Our cohort size is relevant compared to series previously published. The largest cohorts are from the national American database such as the National Cancer Database (NCDB) and the Surveillance, Epidemiology, and End Results (SEER) Program [3,10,20,21,22,28,29], including 1000 to 3500 patients with UCS and/or OCS. Except for these publications, cohorts are usually limited to 250 patients due to the rarity of these tumors [30,31,32,33,34,35,36,37,38,39]. Furthermore, reported cohorts with more than 50 patients for OCS are scarce [14,40,41]. Median age and stage repartition at diagnosis are consistent with data already published [3,10,40,42,43]. Regarding histological analysis, patients included in the network have systematic double pathology reviews, strengthening the quality of our study [24]. The distribution of epithelial component into UCS and OCS are consistent with previous data [44,45,46]. In the sarcoma component, we found more heterologous than homologous both in OCS and UCS. The data available have shown a higher incidence of homologous subtype, but not in all series [44,46,47]. Thus, our study population is representative of the gynecological carcinosarcomas described in the literature. The prognostic value of FIGO stage is established for carcinosarcomas [12,48]. In multivariate analysis, we confirmed that the FIGO stage is correlated with a poor progression-free survival.

In this cohort, the majority of patients underwent primary surgical resection. Complete resection rates were high, suggesting that the surgical quality is related to the network TMRG surgical guidelines. Indeed, it has been reported for other rare tumors, for example ovarian Granulosa cell tumors, that respect of TMRG guidelines improves the quality of surgery [26]. Nearly all OCS received adjuvant chemotherapy, confirming full respect of TMRG guidelines [16,49]. Only one-third of patients with OCS received a combination of chemotherapy and antiangiogenic therapy, probably linked to the approval of bevacizumab in 2012 and reserved for advanced stage (>IIIA).

Therapeutic strategies in the adjuvant setting remains controversial for UCS. Only one really dedicated phase 3 trial was led in this situation [50], but to date, there are no prospective studies indicating that adjuvant radiotherapy or chemotherapy confer an overall survival benefit in patients with gynecological carcinosarcomas. Three drug combinations (cisplatin/ifosfamide, ifosfamide/paclitaxel, and carboplatin/paclitaxel) appear to be effective [50,51,52,53]. Interesting ORR have been reported with various combinations; however, there are no prospective randomized controlled trials that compare the different combination chemotherapy schedules in order to establish the optimal chemotherapeutic regimen sequence. Platinum-based regimens were predominantly used in our study, confirming results from the GOG 261 study [23]. This phase III study comparing carboplatin/paclitaxel to ifosfamide/paclitaxel in women with stage I–IV or recurrent gynecological carcinosarcoma demonstrated that carboplatin/paclitaxel was not inferior to ifosfamide/paclitaxel based on the primary objective OS and was associated with longer PFS [23].

For early stage uterine carcinosarcoma with FIGO stage IA, there is a need for therapeutic adjuvant chemotherapy. In our cohort, adjuvant chemotherapy increased survival outcomes for these patients. Likewise, the large retrospective analysis of American NCDB, which included 2701 stage IA UCS, showed that adjuvant chemotherapy improved overall survival in a multivariate analysis [54]. Based upon these results, adjuvant chemotherapy appears to be beneficial over observation for stage IA UCS.

Moreover, our data seem to be in favor of an adjuvant multimodal treatment for UCS with a longer PFS with sequential chemo-radiotherapy for both stage I/II and stage III/IV. Many retrospective studies suggest that the adjuvant multimodal treatment (combined chemo-radiotherapy approach) is effective, but again, no prospective randomized controlled trials validating this approach has been performed [29,33,55,56].

The best option in a relapse setting is still unknown. Hence, in the first relapse setting, we have shown that a combination of chemotherapy increased ORRs and improved PFS compared to monotherapy. Although they are most often elderly and comorbid patients, these results encourage us not to de-escalate the doublet to a monotherapy. Carboplatin/paclitaxel combination was the most effective systemic therapy, even in pre-treated patients, and should be used for all patients. In the second systemic line/relapse, combinations of chemotherapy also had higher ORRs and longer PFS and should therefore be preferred to monotherapies. The efficacy of anthracyclines remained low, as previously described [57,58]. Patients who received innovative therapies in the first recurrence had improved survival and a good response rate. Although these patients were selected and with good performance status, these data underline the benefit of including patients in clinical trials and the value of the network in providing access to innovation. Currently, the ROCSAN trial (NCT03651206) is dedicated to recurrent carcinosarcomas, testing other therapeutic avenues in this patient population with limited treatment options. This phase II/III trial provides the opportunity to assess the combination of PD-1 and PARP inhibition [59]. Although data have not yet emerged from this study, this combination is a promising treatment regimen. Carcinosarcomas showed high DNA damage response activity, and potentially a high tumor mutation load, resulting in neo-antigens, a synergy between PARPi and anti-PD-1, is expected.

Our study also highlights the value of multidisciplinary consensus issued by experts in the management of gynecological carcinosarcomas. The contribution and the positive impact on the therapeutic management of the national TMRG network was already described for other rare ovarian tumors [26]. The network allows better knowledge of pathologies, and therefore better patient care. This study shows that it is possible to extend the network management to rare tumors of the uterus. The contribution of the pathology network is also an indispensable tool, to encourage slides review, as well as the use of molecular biology assessments, which are not available in all centers. The existence of the TMRG network allows information to be centralized, but also opens up the possibility of dedicated therapeutic trials for patients, such as the ALIENOR study [60] or the ROCSAN trial (NCT03651206), specially for gynecological carcinosarcomas.

This study presents several limitations; we performed a retrospective extraction of clinical data and were faced with missing data, especially related to surgical information, molecular data (such as MSI status and TP53), and histological features. Concerning *BRCA* gene screening, the majority of OCS cases were diagnosed before 2016, and this may explain the low percentage of analysis.

Despite the limitations inherent to retrospective studies, it is essential to analyze all of the cases reported in this type of prospective database in order to identify questions to be asked in future prospective randomized trials.

## 5. Conclusions

This vast cohort study assessed the outcome of patients with carcinosarcomas prospectively recorded in the French Rare Malignant Gynecological Tumors (TMRG) network in a real-life setting. The poor prognosis and the high rate of recurrence of gynecological carcinosarcomas highlight the need for an efficient adjuvant strategy. Interestingly our study suggests the positive impact of adjuvant chemotherapy on survival in all stages, including FIGO stage IA UCS. In the relapse, multidrug regimens increase PFS compared to monotherapy, even in pre-treated patient. Better molecular characterization in the real-life setting should be implemented in order to identify future therapeutic options.

## Figures and Tables

**Table 1:** Patients’ characteristics.

	Primitive Tumor				All Patients	
	Ovarian		Uterine			
	*N* = 112		*N* = 313		*N* = 425	
**Primitive Tumor**						
Ovarian	112	(100.0%)	0	(0.0%)	112	(26.4%)
Uterine	0	(0.0%)	313	(100.0%)	313	(73.6%)
**Age at diagnosis**						
Mean (Std)	66.8 (10.4)		69.1 (9.7)		68.5 (10.0)	
Median (min; max)	67.1 (25.7; 89.9)		69.1 (29.4; 90.2)		68.6 (25.7; 90.2)	
**Age at diagnosis**						
0–59	25	(22.3%)	50	(16.0%)	75	(17.6%)
≥60	87	(77.7%)	263	(84.0%)	350	(82.4%)
**Stage**						
Missing data	6		5		11	
I	9	(8.5%)	140	(45.5%)	149	(36.0%)
II	9	(8.5%)	28	(9.1%)	37	(8.9%)
III	71	(67.0%)	98	(31.8%)	169	(40.8%)
IV	17	(16.0%)	42	(13.6%)	59	(14.3%)
**Detailed stage**						
Missing data	13		27		40	
IA	3	(3.0%)	86	(30.1%)	89	(23.1%)
IB	1	(1.0%)	51	(17.8%)	52	(13.5%)
IC	5	(5.1%)	2	(0.7%)	7	(1.8%)
IIA	6	(6.1%)	4	(1.4%)	10	(2.6%)
IIB	2	(2.0%)	5	(1.7%)	7	(1.8%)
IIC	1	(1.0%)	0	(0.0%)	1	(0.3%)
IIIA	1	(1.0%)	21	(7.3%)	22	(5.7%)
IIIB	4	(4.0%)	9	(3.1%)	13	(3.4%)
IIIC	59	(59.6%)	66	(23.1%)	125	(32.5%)
IV	17	(17.2%)	42	(14.7%)	59	(15.3%)
Metastasis site *						
Missing data	1		0		1	
Liver	7	(43.8%)	5	(11.9%)	12	(20.7%)
Peritoneum	4	(25.0%)	10	(23.8%)	14	(24.1%)
Lung-Pleura	5	(31.3%)	15	(35.7%)	20	(34.5%)
Brain	0	(0.0%)	1	(2.4%)	1	(1.7%)
Extra-pelvic lymph node	4	(25.0%)	5	(11.9%)	9	(15.5%)
Bone	0	(0.0%)	8	(19.0%)	8	(13.8%)
Ovary	0	(0.0%)	3	(7.1%)	3	(5.2%)
Rectum	0	(0.0%)	5	(11.9%)	5	(8.6%)
Bowel	0	(0.0%)	3	(7.1%)	3	(5.2%)
Other	0	(0.0%)	5	(11.9%)	5	(11.9%)
**Histological sarcomatous subtype**						
Missing data	23		56		79	
Homologous	23	(25.8%)	99	(38.5%)	122	(35.3%)
Heterologous	66	(74.2%)	158	(61.5%)	224	(64.7%)
**Histological epithelial subtype**						
Missing data	36		105		141	
Serous	50	(65.8%)	77	(37.0%)	127	(44.7%)
Endometrioid	8	(10.5%)	99	(47.6%)	107	(37.7%)
Other	18	(23.7%)	32	(15.4%)	50	(17.6%)
**Majority component**						
Missing data	55		103		158	
Epithelial	38	(66.7%)	109	(51.9%)	147	(55.1%)
Sarcomatous	19	(33.3%)	99	(47.1%)	118	(44.2%)
**Prior cancer**						
Unknown	2		6		8	
Yes	26	(23.6%)	68	(22.1%)	94	(22.5%)
No	84	(76.4%)	239	(77.9%)	323	(77.5%)
Cancer type *						
Breast	15	(57.7%)	49	(72.1%)	64	(68.1%)
Uterine corpus	1	(3.8%)	0	(0.0%)	1	(1.1%)
Uterine cervix	2	(7.7%)	0	(0.0%)	2	(2.1%)
Lung	0	(0.0%)	2	(2.9%)	2	(2.1%)
Colon	1	(3.8%)	1	(1.5%)	2	(2.1%)
Rectum	0	(0.0%)	4	(5.9%)	4	(4.3%)
Canal anal	1	(3.8%)	5	(7.4%)	6	(6.4%)
Hematologic	1	(3.8%)	3	(4.4%)	4	(4.3%)
Other	5	(19.2%)	7	(10.3%)	12	(12.8%)
**Previous Tamoxifen exposure**						
Unknown	4		14		18	
Yes	2	(1.9%)	20	(6.7%)	22	(5.4%)
No	106	(98.1%)	279	(93.3%)	385	(94.6%)
**History of pelvic radiotherapy**						
Unknown	2		7		9	
Yes	1	(0.9%)	11	(3.6%)	12	(2.9%)
No	109	(99.1%)	295	(96.4%)	404	(97.1%)
**Menopausal hormone therapy exposure**						
Unknown	43		83		126	
Yes	15	(21.7%)	37	(16.1%)	52	(17.4%)
No	54	(78.3%)	193	(83.9%)	247	(82.6%)
***BRCA* status**						
Unknown	72		300		372	
*BRCA*wt	36	(90.0%)	12	(92.3%)	48	(90.6%)
*BRCA1*	1	(2.5%)	1	(7.7%)	2	(3.8%)
*BRCA2*	3	(7.5%)	0	(0.0%)	3	(5.7%)
**Family history of cancer**						
Missing data	21		68		89	
No	34	(37.4%)	132	(53.9%)	166	(49.4%)
Yes	57	(62.6%)	113	(46.1%)	170	(50.6%)
Cancer type *						
Breast cancer	25	(27.5%)	45	(18.4%)	70	(20.8%)
Endometrial cancer	10	(11.0%)	16	(6.5%)	26	(7.7%)
Ovarian cancer	5	(5.5%)	7	(2.9%)	12	(3.6%)
Colorectal cancer	8	(8.8%)	19	(7.8%)	27	(8.0%)
Pancreatic cancer	3	(3.3%)	11	(4.5%)	14	(4.2%)
Prostate cancer	8	(8.8%)	9	(3.7%)	17	(5.1%)
Gastric cancer	1	(1.1%)	1	(0.4%)	2	(0.6%)
Brain cancer	2	(2.2%)	7	(2.9%)	9	(2.7%)
Cutaneous cancer	2	(2.2%)	1	(0.4%)	3	(0.9%)
Other	26	(28.6%)	64	(26.1%)	90	(26.8%)

* A patient may have multiple family history of cancer. One cancer type can therefore be counted several times, and percentages may total higher values than 100%.

**Table 2:** Median PFS (months) by stage in Ovarian Carcinosarcoma.

	Event/Total	Median (95% CI) ^KM^	Hazard Ratio (95% CI) ^Cox^	Survival Estimates (95% CI) ^KM^	*p*-Value
**Stage**					0.0325 *
I–IIA	8/15	24.8 (10.4–NE)	Reference	12 months: 0.73 (0.44–0.89)	
				24 months: 0.52 (0.25–0.74)	
				36 months: 0.44 (0.19–0.67)	
IIB–IV	63/81	12.9 (8.4–19.8)	2.19 (1.05–4.58)	12 months: 0.53 (0.41–0.63)	
				24 months: 0.32 (0.22–0.43)	
				36 months: 0.19 (0.11–0.30)	

^KM^ Kaplan–Meier method; ^Cox^ Cox model; * Logrank test.

**Table 3:** Median PFS (months) by stage in Uterine Carcinosarcoma.

	Event/Total	Median (95% CI) ^KM^	Hazard Ratio (95% CI) ^Cox^	Survival Estimates (95% CI) ^KM^	*p*-Value
**Stage**					<0.0001 *
I	80/139	22.2 (14.9–32.4)	Reference	12 months: 0.70 (0.62–0.77)	
				24 months: 0.47 (0.38–0.55)	
				36 months: 0.41 (0.32–0.50)	
II	23/28	15.1 (8.8–18.3)	1.62 (1.02–2.58)	12 months: 0.61 (0.40–0.76)	
				24 months: 0.25 (0.11–0.42)	
				36 months: 0.21 (0.09–0.38)	
III	68/96	14.8 (11.7–18.7)	1.43 (1.03–1.97)	12 months: 0.60 (0.49–0.69)	
				24 months: 0.31 (0.22–0.41)	
				36 months: 0.27 (0.18–0.37)	
IV	33/35	7.3 (5.5–9.4)	4.24 (2.79–6.44)	12 months: 0.12 (0.04–0.25)	
				24 months: 0.06 (0.01–0.18)	
				36 months: 0.03 (0.00–0.13)	

^KM^ Kaplan–Meier method; ^Cox^ Cox model; * Logrank test.

**Table 4:** Median PFS (months) according to adjuvant strategy in localized Uterine Carcinosarcoma (FIGO Stage I–II) in operated patients.

	Event/Total	Median (95% CI) ^KM^	Hazard Ratio (95% CI) ^Cox^	*p*-Value
**Adjuvant strategy**				<0.0001 *
No adj	19/20	6.5 (2.3–13.5)	Reference	
CT alone	5/7	13.1 (3.1–22.2)	0.54 (0.20–1.45)	
RT alone	53/89	21.0 (14.0–42.6)	0.26 (0.15–0.44)	
Concomitant CT + RT	5/7	18.3 (2.9–NE)	0.34 (0.13–0.93)	
CT then RT	19/41	41.0 (17.0–NE)	0.15 (0.08–0.29)	

No adj: no adjuvant therapy; CT: chemotherapy; RT: radiotherapy. ^KM^ Kaplan–Meier method; ^Cox^ Cox model; * Logrank test.

**Table 5:** Median PFS (months) according to adjuvant strategy in advanced Uterine Carcinosarcoma (FIGO Stage III–IV) in operated patients.

	Event/Total	Median (95% CI) ^KM^	Hazard Ratio (95% CI) ^Cox^	*p*-Value
**Adjuvant strategy**				<0.0001 *
No adj	11/14	3.7 (2.3–5.5)	Reference	
CT alone	33/37	9.4 (5.5–13.5)	0.61 (0.30–1.21)	
RT alone	8/12	10.0 (7.4–NE)	0.33 (0.13–0.84)	
Concomitant CT + RT	4/4	17.4 (6.4–26.1)	0.45 (0.14–1.42)	
CT then RT	29/47	18.9 (14.0–45.6)	0.24 (0.12–0.48)	

No adj: no adjuvant therapy; CT: chemotherapy; RT: radiotherapy. ^KM^ Kaplan–Meier method; ^Cox^ Cox model; * Logrank test.

**Table 6:** Univariate and Multivariate analyses in Uterine Carcinosarcoma (* reference).

Progression-Free Survival				
	Univariate Analysis		Multivariate Analysis	
	Hazard Ratio (95% CI)	*p*-Value	Hazard Ratio (95% CI)	*p*-Value
Age (<60 * vs. ≥60 yr)	1.292 (0.88–1.90)	0.1949		
Stage (II vs. I *)	1.622 (1.02–2.58)	<0.0001	1.595 (1.002–2.537)	0.0024
Stage (III vs. I *)	1.427 (1.03–1.97)		1.087 (0.773–1.529)	
Stage (IV vs. I *)	4.237 (2.79–6.44)		2.204 (1.377–3.527)	
Initial chemotherapy (yes vs. no *)	0.907 (0.69–1.19)	0.4813		
Initial surgery (yes vs. no *)	0.277 (0.17–0.45)	<0.0001		NS
Initial radiotherapy (yes vs. no *)	0.300 (0.23–0.40)	<0.0001	0.357 (0.257–0.496)	<0.0001

**Table 7:** Univariate and Multivariate analyses in Ovarian Carcinosarcoma (* reference).

Progression-Free Survival				
	Univariate Analysis		Multivariate Analysis	
	Hazard Ratio (95% CI)	*p*-Value	Hazard Ratio (95% CI)	*p*-Value
Age (<60 * vs. ≥ 60yr)	1.750 (0.99–3.10)	0.0550		NS
Stage (I–IIA * vs. IIB–IV)	2.189 (1.05–4.58)	0.0374		NS
Initial chemotherapy (yes vs. no *)	0.205 (0.07–0.58)	0.0027	0.147 (0.034–0.635)	0.0103
Initial surgery (yes with CC0 vs. no *)	0.193 (0.09–0.39)	<0.0001	0.179 (0.087–0.368)	<0.0001
Initial surgery (yes with CC1,2,3 vs. no *)	0.667 (0.31–1.42)		0.664 (0.312–1.413)	
Concomitant antiangiogenic (yes vs. no)	0.944(0.57–1.55)	0.8208		

**Table 8:** Response to 1st systemic line/relapse according to chemotherapy regimens. Median durations are expresses in months.

	*N*	Complete Response		Partial Response		Stable Disease		Progressive Disease		Unknown	ORR
Platinum/paclitaxel-based	62	14	(26.4%)	18	(34%)	10	(18.9%)	11	(20.8%)	9	**60.4%**
Platinum/anthracycline-based	27	3	(12.0%)	9	(36.0%)	5	(20.0%)	8	(32.0%)	2	**48.0%**
Platinum-free anthracycline-containing combination	17	1	(6.7%)	6	(40.0%)	3	(20.0%)	5	(33.3%)	2	**46.7%**
Platinum/gemcitabine-based	8	0	(0.0%)	3	(42.9%)	2	(28.6%)	2	(28.6%)	1	**42.9%**
Platinum monotherapy	13	1	(9.1%)	3	(27.3%)	2	(18.2%)	5	(45.5%)	2	**36.4%**
Anthracycline monotherapy	40	2	(6.1%)	4	(12.1%)	3	(9.1%)	24	(72.7%)	7	**18.2%**
Platinum- and anthracycline-free monotherapy	18	0	(0.0%)	0	(0.0%)	3	(23.1%)	10	(76.9%)	5	**0.0%**
Innovative therapy	9	0	(0.0%)	5	(62.5%)	0	(0.0%)	3	(37.5%)	1	**62.5%**

**Table 9:** Progression-free Survival in first relapse according to chemotherapy regimens.

	Event/Total	Median (95% CI) ^KM^	Survival Estimates (95% CI) ^KM^	*p*-Value
				<0.0001 *
Innovative therapy	9/9	8.0 (0.8–10.8)	12 months: 0.11 (0.01–0.39)	
Platinum/anthracycline-based	26/27	8.0 (4.7–10.8)	12 months: 0.29 (0.13–0.46)	
Platinum/paclitaxel-based	49/56	6.8 (4.9–12.0)	12 months: 0.36 (0.24–0.49)	
Platinum/gemcitabine-based	7/8	4.7 (1.2–17.6)	12 months: 0.25 (0.04–0.56)	
Platinum-free anthracycline-containing combination	16/17	3.0 (1.2–6.7)	12 months: 0.12 (0.02–0.31)	
Platinum monotherapy	9/12	2.9 (1.3–14.0)	12 months: 0.23 (0.04–0.52)	
Platinum- and anthracycline-free monotherapy	17/17	2.2 (1.4–4.3)	12 months: 0.06 (0.00–0.24)	
Anthracycline monotherapy	39/39	2.0 (1.7–2.9)	12 months: 0.08 (0.02–0.19)	

^KM^ Kaplan–Meier method; * Logrank test.

**Table 10:** Progression-free Survival in second relapse according to chemotherapy regimens. Median durations are expresses in months.

	Event/Total	Median (95% CI) ^KM^	*p*-Value
			0.0178 *
Platinum/paclitaxel-based	11/11	8.1 (1.6–10.6)	
Anthracycline-free combination	5/6	5.6 (2.3–16.3)	
Anthracycline-containing combination	11/12	3.0 (0.5–5.5)	
Other	16/17	2.9 (1.5–10.3)	
Gemcitabine monotherapy	8/9	2.6 (0.8–3.5)	
Anthracycline monotherapy	17/19	2.1 (1.3–3.3)	
Taxane monotherpy	10/10	1.9 (0.3–4.8)	
Innovative therapy	10/10	1.3 (0.4–2.7)	

^KM^ Kaplan–Meier method; * Logrank test.

**Table 11:** Response to 2nd systemic line/relapse according to chemotherapy regimens (one missing).

	*N*	Complete Response		Partial Response		Stable Disease		Progressive Disease		Unknown	ORR
Platinum/paclitaxel-based	14	2	(20.0%)	6	(60%)	0	(0.0%)	2	(20.0%)	4	**80%**
Anthracycline-free combination	8	0	(0.0%)	4	(57.1%)	1	(14.3%)	2	(28.6%)	1	**57.1%**
Anthracycline-containing combination	12	1	(10.0%)	1	(10.0%)	4	(40.0%)	4	(40.0%)	2	**20.0%**
Anthracycline monotherapy	21	0	(0.0%)	1	(5.9%)	3	(17.6%)	13	(76.5%)	4	**5.9%**
Taxane monotherapy	10	0	(0.0%)	0	(0.0%)	3	(33.3%)	6	(66.7%)	1	**0.0%**
Gemcitabine monotherapy	10	0	(0.0%)	0	(0.0%)	0	(0.0%)	8	(100.0%)	2	**0.0%**
Other	18	0	(0.0%)	1	(7.1%)	4	(28.6%)	9	(64.3%)	4	**7.1%**
Innovative therapy	12	0	(0.0%)	0	(0.0%)	3	(27.3%)	8	(72.7%)	1	**0.0%**

## Data Availability

Data are available on request from the authors.
